# The Effects of Invasive Motor Cortex Stimulation for Neuropathic Pain: Do We See the Full Picture?

**DOI:** 10.1111/papr.70123

**Published:** 2026-02-24

**Authors:** Erkan Kurt, Eline Bijl, Inge Arnts, Suzanne Pouwels, Linda Kollenburg, Jeremy Hanemaaijer, Robert van Dongen, Yvonne Engels, Kris C. P. Vissers, Dylan J. H. A. Henssen

**Affiliations:** ^1^ Department of Neurosurgery Radboud University Medical Center Nijmegen Nijmegen the Netherlands; ^2^ Department of Anesthesiology, Pain and Palliative Medicine Radboud University Medical Center Nijmegen Nijmegen the Netherlands; ^3^ Department of Neurosurgery John Radcliffe Hospital Oxford UK; ^4^ Department of Medical Imaging Radboud University Medical Center Nijmegen Nijmegen the Netherlands; ^5^ Donders Institute for Brain, Cognition and Behavior Radboud University Medical Center Nijmegen Nijmegen the Netherlands

## Abstract

**Introduction:**

Invasive motor cortex stimulation (iMCS) can be a last‐resort treatment of chronic neuropathic pain syndromes. There is evidence that iMCS was found to influence pain intensity, quality of life scores, and pain medication intake in clinical practice. Albeit, qualitative studies published over the last years showed that the expectations and experiences of patients have a significant impact on treatment outcomes. This paper focus on these experiences of chronic neuropathic pain patients treated with iMCS.

**Objectives:**

The aim of this interview study was to map the experiences of patients treated with iMCS in order to evaluate the multidimensional effects of iMCS.

**Materials and Methods:**

Twenty‐eight patients with chronic neuropathic pain treated with iMCS between 2005 and 2018 were interviewed individually. All interviews were semi‐structured using a predefined topic list. An inductive iterative process was performed during the interviews using the constant comparative method. All interviews were audio‐recorded and transcribed verbatim afterwards. Transcripts were coded using direct content analysis. The derived codes were structured into categories and themes. Basic quantitative demographic data were collected, and the decrease in pain intensity after 2 years iMCS was calculated.

**Results:**

Content analysis of the interviews revealed ten relevant categories, which could be structured into three themes: 1. Influence of the iMCS procedure on patients' live; 2. Effects of iMCS on quality of life and participation and 3. Acceptance and satisfaction. In total, 32% (8/25) were identified as responders, and 68% (17/25) as non‐responders. Most patients (71%, 20/28) indicated they would undergo iMCS again. Three patients deceased before the end of the study.

**Conclusion:**

This study suggests that the effects of iMCS should be assessed in a more multidimensional way compared to only the effect on pain as patients confided that the true treatment effect and patient satisfaction were based more on improvements of their quality of life, enabling patients to participate again in society in a meaningful way. In clinical practice, this study emphasizes the need for thorough patient education and realistic expectation management prior to implantation, highlighting both potential benefits and limitations to support informed decision‐making.

## 1 Introduction

Chronic neuropathic pain, affecting approximately 10% of the population [1], has a detrimental impact on all the aspects of life of patients [2, 3, 4]. Therefore, developing new innovative treatments are mandatory using analgesics or interventional treatment options. One of these interventional options is neuromodulation therapy, of which invasive motor cortex stimulation (iMCS) is still considered to be a more experimental treatment option. iCMS is first described in 1991 [5] and implies that neurosurgeons place an intracerebral extradural electrode to apply subthreshold stimulation over the primary motor cortex in order to alleviate continuous (neuropathic) pain in the homologous body part. Although the exact mechanism of action remains elusive [6], there is evidence that iMCS significantly reduces pain intensity scores in approximately 60% of cases [7, 8, 9, 10]. Also, iMCS was found to improve quality of life, as measured by questionnaires and scoring systems [11]. However, in recent years, it appears that the perspectives and experiences of patients treated with iMCS have never been investigated in depth for this treatment option.

Over the past years, it has been reported that patient perspectives and experiences are crucial in the development of dedicated treatment paradigms and contribute to a better personalized evaluation and improvement of specific pain treatment strategies [12, 13]. Qualitative studies showed that this type of research is capable of providing a detailed insight into the subjective personal perspectives and individual experiences of each patient. As qualitative data of patients' perspectives and experiences are currently lacking [14, 15], we only have a limited understanding of the spectrum of the multidimensional effects of iMCS. Therefore, this study aims to gain more in‐depth insights into the effects of iMCS on daily life and experience, by use of interviewing patients after having undergone iMCS treatment for chronic neuropathic pain.

## 2 Materials and Methods

### 2.1 Study Design and Participants

To be eligible to participate in this study, participants needed to suffer from chronic neuropathic pain and must have undergone iMCS surgery at least 2 years before start of this study. Patients could participate regardless whether the iMCS system was still implanted. Patients were excluded if their cognition was impaired or if they had no full comprehension of the Dutch language.

All patients who underwent surgery in the period 2005–2018 were contacted by phone by their medical specialist to inform them about this study. After this phone call, all patients who considered to participate received an information letter. After informed consent was obtained, all participants were contacted to schedule individual interviews. It was decided to interview all participants because it remains uncertain as to how many interviews are required to accomplish a saturation of the topic‐list.

All interviews were performed by an independent researcher (E.B.) who had no professional or personal relationship with the patients. Interviews were taken by phone between June and August 2020, due to the ongoing COVID‐19 pandemic. Interviews were semi‐structured by use of a topic list. The topic list was based on previous research experiences with regard to qualitative research, scientific literature on qualitative research in pain medicine, and clinical experience of the research team. Three overarching questions were used as anchoring points: (1) Would you accept again to undergo iMCS treatment if the same outcome was guaranteed as you have now and explain why?; (2) How has iMCS impacted your daily life?; and (3) How do you think your life would change if the iMCS system was not in place anymore?

Further, we collected basic quantitative patient characteristics (Table 1). Responders of iMCS were defined as having ≥ 30% pain relief on a visual analogue scale (VAS) [16].

**TABLE 1:** Patient characteristics of the eligible participants treated with iMCS.

#	Sex	Age (years)	Duration of pain (years)	Pain syndrome	Location of pain targeted with iMCS	VAS pre‐operative	VAS after 2 years	VAS decrease (%)	Responder	Would undergo iMCS again
1	M	56	12	Trigeminal deafferentation	Hemiface	90	5	94.4	Yes	Yes
2	F	56	4	CPSP	Upper limb + hemiface	80	15	81.3	Yes	Yes
3	F	67	26	Wallenberg Syndrome	Hemiface	97	34	65.0	Yes	Yes
4	M	49	32	BPA	Upper limb	85	42	50.6	Yes	Yes
5	M	61	16	Wallenberg Syndrome	Hemiface	100	51	49.0	Yes	Unsure
6	M	57	10	Trigeminal deafferentation	Hemiface	84	50	40.5	Yes	Yes
7	M	42	10	Trigeminal deafferentation	Hemiface	62	42	32.3	Yes	Yes
8	M	74	2	CPSP	Upper limb	75	51	32.0	Yes	Yes
9	M	48	17	BPA	Upper limb	100	74	26.0	No	Unsure
10	F	48	17	Wallenberg Syndrome	Hemiface	100	75	25.0	No	Yes
11	F	59	9	CPSP	Upper limb	73	56	23.3	No	Yes
12	F	63	5	Atypical facial pain with history extraction of the submandibular gland	Hemiface	82	71	13.4	No	Yes
13	M	64	7	CPSP	Upper limb + hemiface	88	78	11.4	No	Yes
14	M	63	7	Wallenberg Syndrome	Hemiface	71	63	11.3	No	No
15	F	70	2	Trigeminal deafferentation	Hemiface	88	79	10.2	No	Yes
16	F	57	6	CPSP	Upper limb	76	70	7.9	No	Yes
17	M	55	12	CPSP	Hemiface	65	60	7.7	No	Yes
18	F	54	5	CPSP	Hemiface	60	58	3.3	No	Yes
19	M	68	3	BPA	Upper limb	66	65	1.5	No	No
20	F	42	9	Trigeminal deafferentation	Hemiface	80	80	0.0	No	Yes
21	F	46	5	CPSP	Upper limb	56	56	0.0	No	Unsure
22	F	54	5	Trigeminal deafferentation	Hemiface	67	70	−4.5	No	Yes
23	M	68	7	Wallenberg Syndrome	Hemiface	32	36	−12.5	No	No
24	F	45	3	Postherpetic Neuralgia	Hemiface	65	78	−20.0	No	Yes
25	F	55	9	Trigeminal deafferentation	Hemiface	53	72	−35.9	No	Unsure
26	F	68	2	CPSP	Upper limb	91	N/A	N/A	N/A	Yes
27	M	64	1	CPSP	Hemiface + hemibody	65	N/A	N/A	N/A	Unsure
28	M	66	5	Wallenberg Syndrome	Upper limb	77	N/A	N/A	N/A	Yes

Abbreviations: BPA, brachial plexus avulsion; CPSP, central post stroke pain; iMCS, invasive motor cortex stimulation; N/A, not available; VAS, Visual Analogue Scale.

### 2.2 Data Analysis

An inductive, iterative process was applied during the interviews using the constant comparative method. Data analysis began immediately after completing the first interview. Codes generated from each previous interview served as a foundation for coding the subsequent one, with additional codes introduced as needed. When unclear, additional questions were asked to ensure proper understanding of the participants' meaning. Interviews where audio‐recorded and transcribed verbatim. Field notes were made during the interview.

Transcripts were analysed using inductive content analysis using Atlas.ti software (https://atlasti.com/; *version 8.4.20.0*). Code names were assigned to specific features within the transcribed interviews, using the derived codes as the starting point for the next interview and adding new codes when appropriate. Coding of the first five interviews was carried out by two researchers independently (E.K. and E.B.). In case of disagreement, a third researcher (D.H.) independently reviewed the codes and served as an adjudicator. During a group meeting, all codes were categorized into categories and further into themes, while checking the original transcript for adequate interpretation (D.H, E.B, E.K.).

### 2.3 Ethical Considerations

The study was performed according to Good Clinical Practice guidelines. The Medical Review Ethics Committee concluded that this study was not subject to the Medical Research Involving Human Subjects Act. In addition, all participants provided written informed consent to participate in this qualitative study.

## 3 Results

A total of 41 patients with refractory pain syndromes to conventional pain treatments were implanted iMCS. Of these, 28 agreed to participate in this qualitative interview study. In three patients, VAS data were not available after 2 y post iMCS because they deceased before the end of the study. An overview of the included patients is provided in Table 1. In total, 32% (8/25) were identified as responders and 68% (17/25) as non‐responders. Most patients (71%, 20/28) indicated they would undergo iMCS again.

**FIGURE 1:**
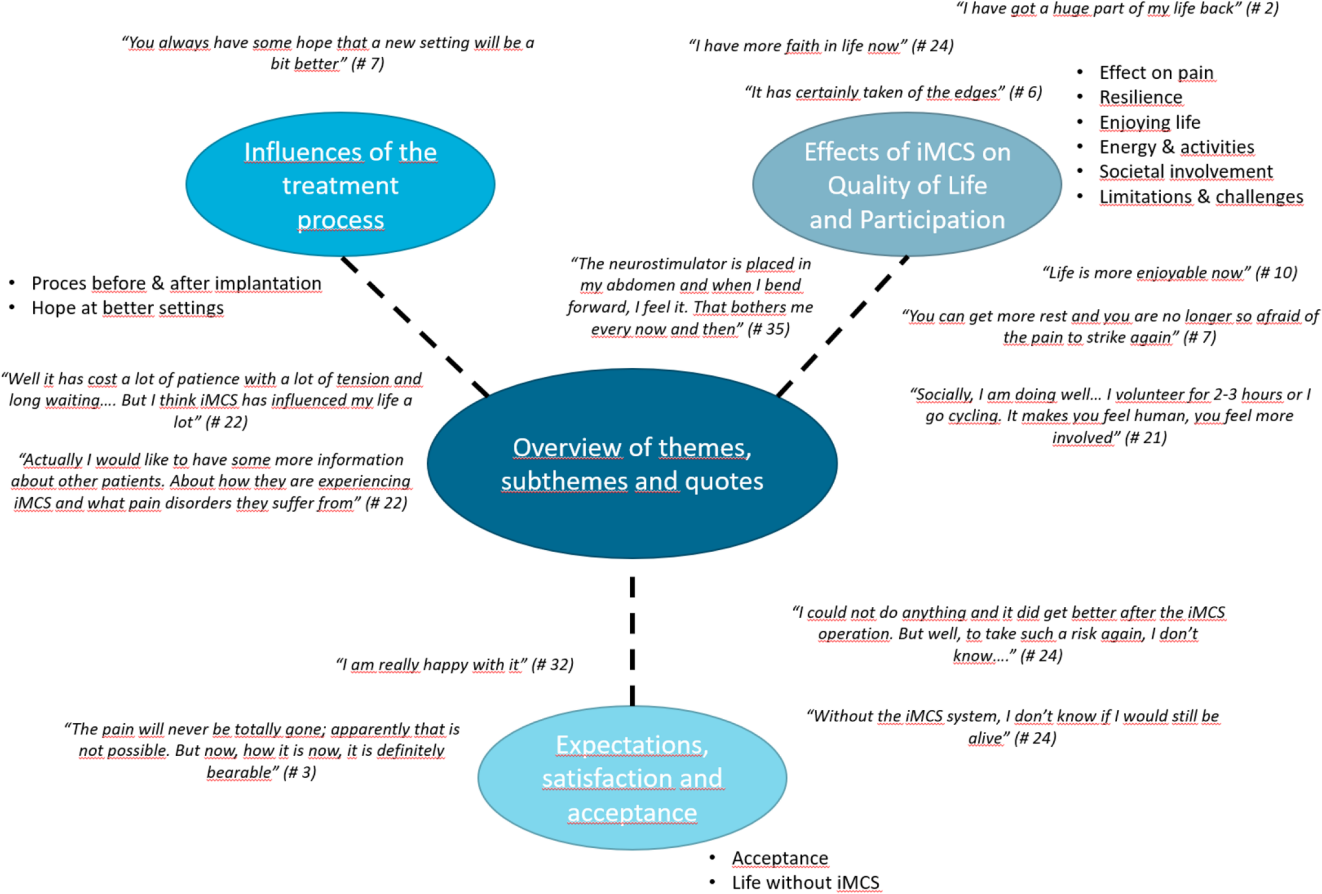
Overview of themes, subthemes, and quotes.

Interviews lasted up to 40 min. Analysis of the interviews revealed three themes, which were categorized into ten subthemes, which are illustrated in Figure 1.

### 3.1 Influence of the iMCS Process on Patients' Lives

**Process before and after implantation**: In total, 84% (21/25) of patients reported that the extensive preoperative diagnostic process leading up to iMCS implantation (workup includes functional MRI investigation, somatosensory‐ and motor‐evoked potential tests, multiple questionnaires, and neuropsychological testing) felt as a recognition of their pain syndrome. In contrast, 16% (4/25) patients reported that this process was rather bothersome as it causes fatigue (12%, 3/25) and delay in planning of surgical procedure (4%, 1/25). The surgical procedure was generally experienced as manageable. All patients were positive about the support of the medical team, although they also disclosed that they would like to have had more contact with fellow iMCS patients, both prior to the surgical intervention and after implantation.

**Hope at better settings**: After the implantation, the process of finding optimal stimulation parameters was mentioned commonly as a resource of hope. However, trying new settings more often resulted in disappointment and uncertainty around the outcome of iMCS. This had a big impact on patients' lives as during these months, patients focused more on their pain during this period of time. This increased focus on their pain led to a consequent increase in pain.

### 3.2 Effects of iMCS on Quality of Life and Participation

**Effect on pain**: Most patients experienced a substantial pain relief after implantation, and iMCS took away the most intense peaks often without affecting the background pain. Although patients still experienced pain, they disclosed that their pain became more bearable and acceptable. However, this “background pain” still had a significant impact on patients' lives. Patients also revealed that when high levels of pain intensity decreased—even just slightly—this was highly valued. Therefore, the majority of interviewees mentioned that, in their opinion, pain intensity scores could not be considered a representative measure of their complex pain experience. Some patients became aware of the effect of the stimulation when they by mistake had turned off the system. Effect of stimulation was usually stable and could be increased again with adapting the stimulation settings or switching the system “off” and “on” again.

**Resilience**: All interviewed patients expressed that they experienced that using iMCS helped them cope better with their pain. This also helped them improve their dealing with major life events. Fear and panic attacks and consequent need for psychological support decreased over time due to the iMCS.

**Enjoying life**: In general, patients expressed to enjoy life more and experienced an improved QoL. Besides, they felt more satisfied, and they were more at ease with themselves. However, those patients that experienced substantial pain had difficulties to stay positive about their live.

**Energy and activities**: Patients experienced an increase in energy and a greater desire to participate in daily activities. They took more initiative, slept better, and spent less time in bed during the day. Their capability to participate in various (physical) activities like housekeeping, gardening, walking, cycling, and driving increased; and this was often worth have a timely increase in their pain. Moreover, patients were less restricted by pain‐provoking factors.

**Societal involvement**: Due to a decrease in pain intensity in combination with a decrease in daily pain medication intake, patients told to be more present and alert. This improved their relationships with family and friends. Also, increased capability to handle crowds had a positive influence on the ability to participate in social activities. A minority of the patients (16%, 4/25) were able to resume occupational as well as voluntary work, often with adjustments, restoring a sense of meaning and involvement in society.

**Limitations and challenges**: for most of the patients, pain still limited to some extent their daily activities and functionality. This helped them explore and respect their personal boundaries, and some still needed day‐to‐day supportive care. Moreover, several patients suffered from other pain syndromes than the neuropathic pain treated with iMCS or were restricted by comorbidities. Although most patients did not experience technical problems, the iMCS system itself caused stress to some. It caused worries, about empty batteries or the electrode moving, tingling sensations, or headache with stimulation and movement restrictions by the abdominal battery.

### 3.3 Acceptance and Satisfaction of Patients After iMCS

**Acceptance**: Patients felt increased ability to accept their current situation having less pain or realized they tried all current available medical treatments. So, being at peace with the results involved that they were also accepting the limits of this treatment. Most patients described having clear realistic expectations that full pain relief was not possible. Despite the fact that their pain relief was less than expected, they were satisfied with the current effect and reaching the preoperative goal.

**Life without iMCS**: Majority of the patients (72%, 18/25) would choose again for iMCS, and they would recommend it to others. The idea of living without iMCS made many patients feel uncertain about their capability to handle the pain. Thinking about returning to the preoperative situation invoked feelings of hopelessness, uselessness, and meaninglessness. Several patients stated to consider ending their lives if they had to live without iMCS again. Although some patients were disappointed of their result and felt that their iMCS treatment wouldn't make any difference on their pain experience or daily living.

## 4 Discussion

In this qualitative study, patients with severe chronic neuropathic pain who were treated with iMCS disclosed three important themes: 1. Influence of the iMCS process on patients' lives; 2. Effects of iMCS on quality of life and participation; and 3. Acceptance and satisfaction of patients treated with iMCS who are living with chronic neuropathic pain. Taken altogether, these three themes summarize the clinical complexity of iMCS for both patients and the multidisciplinary team of health care professionals and suggest that chronic neuropathic pain, as a multidimensional problem, should not be evaluated by use of quantitative assessments and outcome parameters alone. *I*n this study, outcomes on pain intensity as measured by the VAS varied substantially. While several patients achieved marked reductions in pain (e.g., decreases of > 80%), the majority experienced more modest changes, and in some cases, VAS scores remained stable or even worsened. Overall, only a subset (32%, 8/25) met the conventional responder criterion of at least 30% pain reduction. These findings underline the limited reliability of VAS as a sole measure of treatment success in neuromodulation. Despite modest changes in pain intensity, most of the patients (71%, 20/28) still indicated they would undergo iMCS again, reflecting perceived benefits in domains beyond pain relief.

To the best of our knowledge, this is the first qualitative study that evaluates patient experiences in long‐term post‐operative iMCS. A key finding of the study was that most interviewees expressed that pain intensity scores did not adequately represent their complex experience of pain. Although reductions in pain intensity were modest, several patients reported perceived benefits in terms of functioning, sense of control, and daily participation, suggesting that neuromodulation may provide value beyond analgesic effects. At the same time, improvements in quality of life were not consistently observed, which aligns with earlier reports in chronic pain populations. These observations point to the importance of considering multidimensional outcomes when evaluating neuromodulation since patients may still prefer treatment even in the absence of substantial pain reduction.

In a previously published letter to the editor the authors showed that a patient's global impression of change and face‐to‐face interviews revealed subtle treatment effects of repetitive transcranial magnetic stimulation of the primary motor cortex to treat chronic neuropathic orofacial pain. These effects were overlooked when only quantitative results were taken into account [17]. Moreover, the importance in neuromodulatory therapy for severe chronic neuropathic pain of incorporating qualitative data apart of quantitative data in outcome assessment has been reported frequently [18, 19, 20, 21].

The phenomenon that most patients are willing to undergo iMCS treatment again even if there was only a limited effect on their pain intensity scores and has also been described by André‐Obadia et al. [22]. In their study, 20 patients who underwent iMCS were included and followed for a maximum of 9 years. Although their study focused on quantitative outcomes, the results highlighted that pain relief after iMCS was not correlated with an improvement of other important quality of life dimensions. When asked whether patients would be willing to be operated again, the patient response appeared to be related mostly to the improvement of their quality of life scores [22]. Interestingly, Andre Obadia et al. showed that the willingness to be operated again did not correlate with their pain intensity scores [22]. In line with this, Nuti et al. described that of 31 patients, the majority would be willing to undergo iMCS surgery again, despite a moderate percentage of pain relief (10%–39%) [23].

Recently, measuring the effectiveness with goal‐identification has been proposed for spinal cord stimulation to treat chronic neuropathic pain [20]. This might be a valuable addition to enrich the assessment of the iMCS outcome as well. Moreover, the qualitative findings of another study provided insights into patients' experiences of their treatment journey. Themes of patients' experiences with iMCS seem to be similar to themes that arose from other qualitative studies on neuromodulation treatments [24]. Interestingly, the desire to share experiences with fellow patients was found in other neuromodulation research as well [25] and might indicate an opportunity to improve personalized patient‐centered care. Moreover, even though most of our patients stated to have no expectations of full pain relief, a key factor in satisfaction seemed to be acceptance of the limitations of the effects if iMCS, which is similar to other neuromodulation treatments [24]. Since acceptation improves quality of life in neuropathic pain patients [26], goal identification and management of expectations should be addressed during iMCS treatment.

Finally, it should be emphasized that although positive responses were reported for a subset of participants in this study, the efficacy varies between patients and depends on a complex interplay of both quantitative outcomes (VAS) and qualitative outcomes.

### 4.1 Strengths and Limitations

Multidimensional assessments have been implemented for evaluating responses to various treatments for chronic pain. Nevertheless, the efficacy of iMCS is often examined using a unidimensional approach, solely focusing on quantitative, but not qualitative outcome measures. Consequently, a questionable perception of responses to iMCS arise, which unfortunately hinders formal recognition of this therapy and results in it still being considered an experimental treatment, despite promising outcomes and research ongoing for over 30 years. For this matter, this article can be considered highly unique, as it is the first describing use of a multidimensional approach for iMCS in patients with chronic pain. The interviews provided a rich insight in patients' experiences and increased transferability and reliability of the findings.

Telephone interviews might be inferior to face‐to‐face interviews [27], but previous research showed that interviews conducted by telephone are valuable for collecting data and might even allow participants to disclose sensitive information more freely [28]. It should be noted that attrition bias may have affected the outcomes of this study as patients with negative responses to iMCS may have been less likely to participate in the interviews.

Finally, it should be noted that a considerable amount of time elapsed between receival of iMCS and the interview. As a consequence, habituation to the iMCS effects and recall bias may affect the outcomes of this study.

## 5 Conclusion

The results of this qualitative study show that iMCS may have a multidimensional impact, meaning that both quantitative and qualitative aspects are affected, on the lives of patients suffering from neuropathic pain. Patients disclosed that reducing the most violent pain is of great value and improves substantially their quality of life by enabling them to participate again in social engagement and enjoy their day‐to‐day life. In clinical practice, this emphasizes the need for thorough patient education and realistic expectation management prior to implantation, highlighting both potential benefits and limitations to support informed decision‐making.

## Author Contributions

All authors have made a substantial contribution to the work and approved it for publication. All authors provided input to the revised version. Erkan Kurt: first author, data acquisition, design. Eline Bijl: data acquisition. Inge Arnts: data acquisition. Suzanne Pouwels: data acquisition. Linda Kollenburg: data acquisition and illustration. Jeremy Hanemaaijer: data acquisition. Robert van Dongen: analysis. Yvonne Engels: interpretation of data. Kris C.P. Vissers: analysis and interpretation of data. Dylan J.H.A. Henssen: data acquisition, design, and interpretation of data.

## Funding

The authors report no funding sources.

## Ethics Statement

We confirm that we have read the Journal's position on issues involved in ethical publication and affirm that this report is consistent with those guidelines.

## Consent

All patients approved for the anonymised use of their data for this manuscript. The study was performed according to the Good Clinical Practice guidelines. The Medical Review Ethics Committee concluded that this study was not subject to the Medical Research Involving Human Subjects Act. In addition, all participants provided written informed consent to participate in this qualitative study.

## Conflicts of Interest

The authors declare no conflicts of interest.

## Data Availability

The data used and analyzed in this study are all available in the current manuscript.
